# Abnormal macrophage response to microbial stimulus in a 43-year-old man with a severe form of atherosclerosis: a case report

**DOI:** 10.1186/1752-1947-4-183

**Published:** 2010-06-18

**Authors:** Maria Conti, Francesca Sanna, Giulia AM Farci, Sabrina Uda, Giovanna Porcu, Maria Collu, Rosa R Bonatesta, Barbara Batetta

**Affiliations:** 1 Department of Cardiovascular and Neurological Sciences, University Hospital, 09042 Monserrato, Italy; 2 Department of Biomedical Sciences and Technologies, Institute of Experimental Pathology, Via Porcell, 09124 Cagliari, Italy; 3 Department of Medicine, University Hospital, 09042 Monserrato, Italy; 4 Department of Neuroscience, University Campus, 09042 Monserrato, Italy

## Abstract

**Introduction:**

New evidence indicates infections are emerging as risk factors for atherosclerosis although their specific role in the development and progression of atherosclerosis is still unclear.

**Case presentation:**

A 43-year-old Caucasian man who had been treated for four years for multiple sclerosis progressively manifested systemic hypertension, polycythemia, peripheral arterial occlusion with intermittent claudication, and persistent headaches. In 2006, an instrumental analysis (magnetic resonance imaging) of our patient revealed widespread fibrocalcific atherosclerotic lesions which accounted for all his current symptoms, including those related to microbial stimulus. Two particular aspects were of interest, namely a lack of conventional cardiovascular risk factors and a negative family history for cardiovascular events. His chemical blood tests all yielded negative findings although a low positive hepatitis C virus-ribonucleic acid titer was detected. The titer had progressively increased and worsening atherosclerosis threatened the life of our patient. Interferon therapy was not appropriate for our patient due to the severe adverse effects observed shortly after its administration.

**Conclusions:**

The reaction of individual cells to infections may provide an explanation as to why individuals with a similar microbial burden, corrected for the presence of other risk factors, display a different susceptibility to developing or worsening atherosclerosis. The identification of susceptible individuals and the treatment even of silent infections may provide an additional tool against atherosclerosis and its clinical complications. The evaluation of cell susceptibility before and after the correction of risk factors may contribute to the assessment of the efficacy of drug therapy.

## Introduction

Atherosclerosis is the leading cause of death in the majority of industrialized countries. The absence of "traditional" risk factors and the ready availability of new therapeutic options are not sufficient to provide overall protection against disease and its complications. Every effort is being made by the scientific community to identify conditions leading to disease of the arterial wall and to find new diagnostic procedures for identifying susceptible individuals. New lines of research have begun to show evidence to indicate that infectious agents are emerging as risk factors, although their role in the development and progression of atherosclerosis is still unclear.

Association of infections with atherosclerosis was first reported in the 1970s [1]. Over the past decade awareness of the possible association between atherosclerosis and certain persistent bacterial and viral infections has steadily increased [2-6]. Numerous reports have referred to the contributory role in the atherosclerotic process played by periodontal diseases, which have often been associated with myocardial infarction and cerebrovascular events [7]. However, this association does not necessarily prove the existence of atherogenic effects, particularly in view of the widespread distribution of the microorganisms involved.

Several authors have hypothesized that the number of different pathogens to which an individual has been exposed might promote a synergistic inflammatory response capable of exacerbating atherosclerosis [7-9].

A case of severe atherosclerosis in a 43-year-old patient who had no conventional risk factors, but with a known hepatitis C virus (HCV), infection and an abnormal immune cell response to infective stimuli, provided us with the opportunity to re-examine the impact of infections on cardiovascular diseases. In fact our case suggests that in patients with a similar microbial burden, but displaying a different susceptibility to atherosclerosis even in the presence of other risk factors, individual immune cell response to infective stimulus must be taken into account.

## Case presentation

A 43-year-old Caucasian man was referred to the Department of Internal Medicine at the University Teaching Hospital in Cagliari. His clinical manifestations started in 2002 with a sudden onset of neurological symptoms. Magnetic resonance imaging (MRI) of lesions was consistent with a diagnosis of multiple sclerosis. Our patient, employed as a prison officer, was not deemed fit for work. In 2003, he was admitted to a small community hospital where he was also diagnosed with hypertension, polycythemia and intermittent claudication. Finally, in 2006, he was referred to the Multiple Sclerosis Centre at Cagliari University where a vascular origin was postulated for his neurological symptoms. Tests carried out on fibrocalcific atherosclerotic lesions accounted for the concomitant presence of systemic hypertension, polycythemia, brain injury and peripheral arterial occlusion. His electrocardiogram (ECG) findings were normal, although due to a compromised limb function, an exercise ECG could not be performed. Ethical reasons also dissuaded us from performing a coronary angiograph.

**Figure 1:**
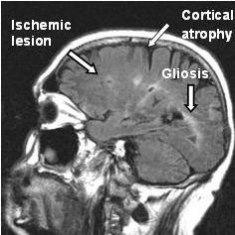
**MRI of our patient's brain**. Mild cortical atrophy with bilateral lacunar ischemic lesions and gliosis in the cerebral white matter, mainly in the semi-oval center and the corona radiata.

**Figure 2:**
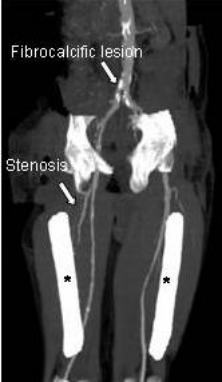
**Abdominal computed tomography angiography of our patient's aorta and lower limbs**. Widespread fibrocalcific atherosclerotic lesions are present. Both renal arteries and iliac branches are affected by severe stenosis (70% to 85%). Tibial arteries are occluded, thus preventing the examination of parts of the legs.

His family history for cardiovascular events was negative. A brain MRI revealed mild cortical atrophy with bilateral lacunar ischemic lesions and gliosis in his cerebral white matter, mainly in the semi-oval center and the corona radiata (Figure 1). The presence of extensive arterial damage with bilateral atherosclerotic lesions was revealed using color Doppler ultrasound. Abdominal computed tomography (CT) angiography of his aorta and lower limbs showed widespread fibrocalcific atherosclerotic lesions (Figure 2). Both his renal arteries and iliac branches were affected by severe stenosis (70% to 85%). His tibial arteries were occluded, preventing us from examining parts of his legs. A plaque determining a 50% reduction in his left carotid gauge was also revealed using color Doppler ultrasound. Blood tests showed normal serum levels for lipids and glucose, and his systemic inflammatory markers were all within normal ranges (Table 1). Common thrombophilic conditions and markers of immunological diseases all yielded negative findings. Infection markers for human immunodeficiency virus (HIV), toxoplasmosis, herpes I, II, hepatitis B surface antigen (HBsAg) were also negative, but a low titer of HCV-ribonucleic acid (RNA) was found.

**Table 1:** Clinical characteristics of our patient.

Total cholesterol (mmol/L)	5.23	ESR (mm/h)	6
LDL-C (mmol/L)	2.59	Fibrinogen (μmol/L)	10.29
HDL-C (mmol/L)	1.76	IL-1β (pg/mL)	2.3 (n.v. < 3.9)
ApoA1 (g/L)	1.48	TNF-α (pg/mL)	9.5 pg/mL (n.v. < 15.6)
ApoB (g/L)	0.92	IL-6 (pg/mL)	0.5 ( < 3.13)
Triglycerides (mmol/L)	152	Leukocytes (mm^3^)	8.500
Lp(a) (mg/L)	37	HCV-RNA (IU/mL) genotype 1b	396.000
Glucose (mmol/L)	4.9	Ab anti-CMV IgG (IU)	5.6 (n.v. < 0.4)
Homocysteine (μmol/L)	12.49	Ab anti-CMV IgM (IU/mL)	8 (n.v. < 15)
CRP (mg/ml)	6.5(n.v10)		

CMV: cytomegalovirus; CRP: C-reactive protein; ESR: erythrocyte sedimentation rate; HCV: hepatitis C virus; IL: interleukin; Lp(a): Lipoprotein a; n.v.: normal values; TNF-α: tumor necrosis factor alpha.

**Figure 3:**
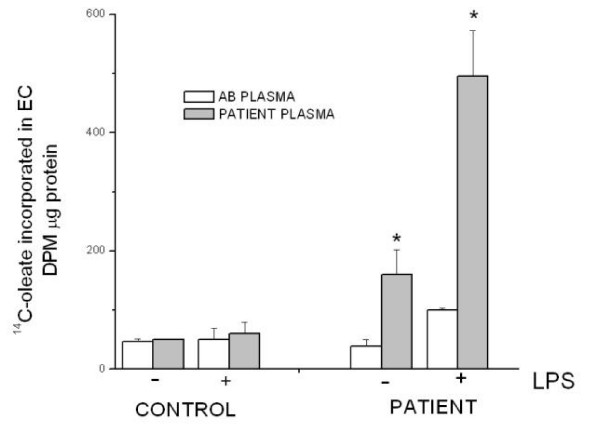
**In this experiment, following the removal of non-adherent cells, cells from our patient and controls were grown in RPMI 1640 media, supplemented with 10% patient plasma, while others cells were cultured in RPMI 1640 supplemented with 10% AB serum**. The cells were subsequently treated for 48 hours as depicted in the figure. Four hours before harvesting, the cells received 74 KBq/mL of ^14C-oleate. After incubation the cells were washed with ice-cold phosphate-buffered saline (PBS), and lipids were extracted with acetone. Neutral lipids were then separated and determined as described in material and methods (Additional File 1). Each value represents the mean ± SEM of five separate experiments (P < 0.05 versus the corresponding control)

**Figure 4:**
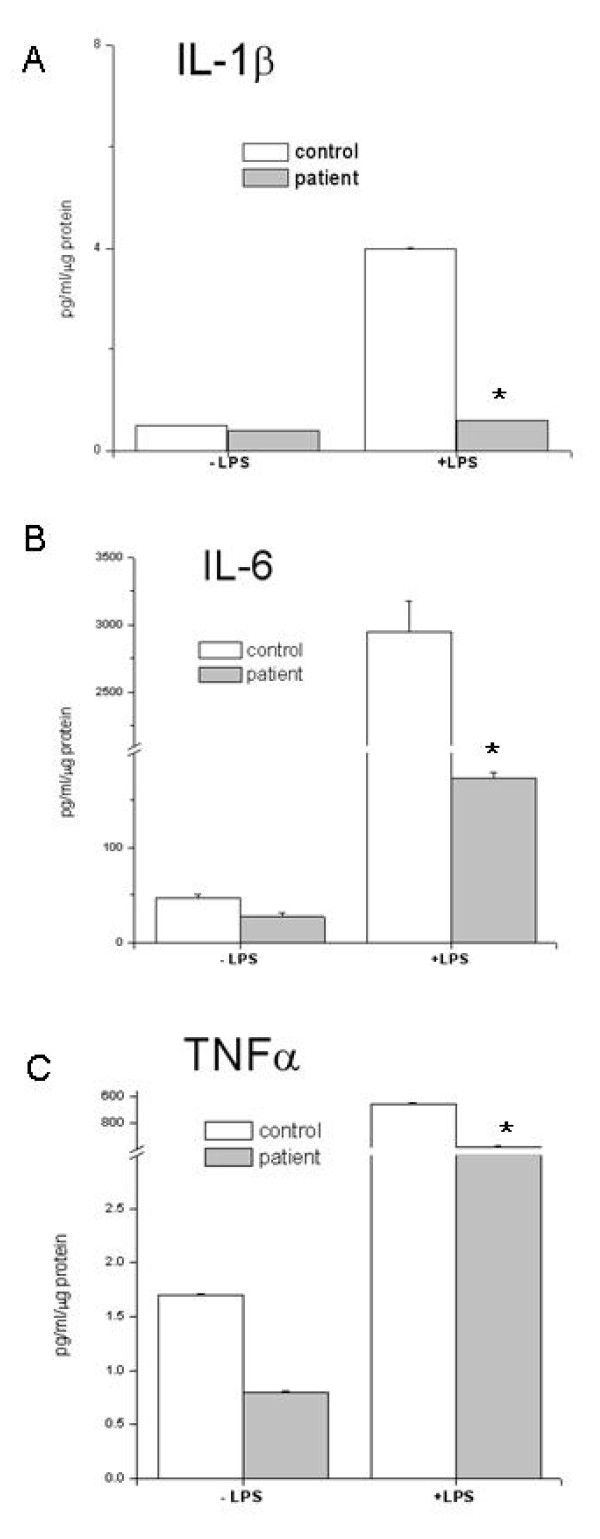
**On day nine the macrophage monolayer was washed, fresh culture medium was supplied, and cells cultured as depicted in the figure for a further two days in the presence of lipopolysaccharide**. Cytokine secretion into the medium was evaluated on day 11. Each value represents the mean ± SEM of five separate experiments (P < 0.05 versus the corresponding control).

As our patient showed negative results for conventional risk factors for atherosclerosis, we considered the possibility that the disease was related to HCV infection. However, no virus was detected in his lymphocytes, monocytes and macrophages. These findings, together with a suspected abnormal response to infective stimuli, prompted us to investigate our patient's immune cell reactivity. Lipopolysaccharide (LPS), a Gram-pathogen considered to be a potential contributor to the development of atherosclerotic plaque even at extremely low serum concentrations [10,11], was used to evaluate his macrophage response. An abnormal reactivity of macrophages to LPS, exacerbated by our patient's own serum, was found; in fact this reaction mainly caused foam cell formation (Figure 3) rather than pro-inflammatory cytokine production (Figure 4). Neither LPS nor our patient's serum were capable of reproducing similar effects on macrophages in healthy subjects. In addition to the role played by viruses and other unknown factors, these findings suggested the possibility that our patient's macrophages were unusually susceptible to infective stimuli.

Our patient was prescribed the following: interferon-gamma therapy was suspended to avoid its adverse effects; atenolol 10 mg/day; amlodipine 10 mg/day; cardio-aspirin 100 mg/day; lansoprazole 30 mg/day; allopurinol 300 μg/day; escitalopram 10 mg/day; and therapeutic bleeding about once a month.

HCV mRNA was first detected in 2006, the titer progressively increased but the criterion for interferon therapy was only reached in 2007. Unfortunately, the therapy was withdrawn because of the severe adverse effects observed shortly after its administration (aplastic myelosis, behavioral disturbance with depression, neurasthenia and anorexia). As our patient's lipids and systemic inflammatory markers were normal, it was not possible to prescribe conventional therapy to counteract the progression of his atherosclerosis. The disease is still worsening (the arterial tree has the appearance of being coated with wax) concomitantly with the increase in his HCV titer.

## Discussion

Despite an increasing number of studies performed to investigate the association between infections and vascular diseases, no consensus has been reached to date on the possible atherogenic effects of infectious agents. The pathogenetic mechanisms underlying this disease remain unclear, and the associations revealed do not necessarily prove the existence of atherogenic effects, particularly in view of the widespread distribution of the microorganisms involved.

This case report suggests that in susceptible individuals the presence of a chronic infective stimulus may directly activate metabolic pathways (such as cholesterol esterification) leading to foam cell formation and thus to atherosclerosis. Furthermore, it also implies that this condition may be exacerbated by acute infections, as is suggested by the exceedingly high rate of cholesterol esterification found in patient macrophages incubated with its own plasma plus LPS, but not found in control cells. Thus, in patients with a similar microbial burden who display a different susceptibility to atherosclerosis, even in the presence of other risk factors, individual immune cell response to infective stimulus should be taken into account.

To the best of our knowledge this is the first case that describes the molecular mechanisms by which macrophages respond to infective stimuli inducing foam cell formation. We suggest that research on silent infections and the evaluation of immune cell reactivity to microbial injuries, could lead to the development of an additional strategy for preventing and controlling atherosclerosis.

## Conclusions

The reaction of individual cells to infections may provide an explanation as to why individuals with a similar microbial burden, corrected for the presence of other risk factors, display a different susceptibility to developing or worsening atherosclerosis. The identification of susceptible individuals and the treatment of silent infections may provide an additional tool against atherosclerosis and its clinical complications. The evaluation of cell susceptibility before and after the correction of risk factors may contribute to the assessment of the efficacy of drug therapy.

## Abbreviations

ECG: electrocardiogram; HCV: hepatitis C virus; LPS: lipopolysaccharide; MRI: magnetic resonance imaging.

## Competing interests

The authors declare that they have no competing interests.

## Authors' contributions

MC and GAMF analyzed and interpreted our patient's data. FS, SU, GP and RRB performed experimental work. MC and BB were the primary drafters of the manuscript. All authors read and approved the final manuscript.

## Consent

Written informed consent was obtained from the patient for publication of this case report and any accompanying images. A copy of the written consent is available for review by the Editor-in-Chief of this journal.

## Supplementary Material

Additional file 1 **Materials and methods**. The data provided discuss the materials and methods used in plasma preparation, clinical chemistry, cell isolation and culture, analysis of cholesterol-laden macrophages, cell protein isolation and western blotting analysis, cytokine assay, cholesterol esterification, and statistical analysis. Click here for file

## References

[B1] Fabricant CG Fabricant J Litrenta MM Minick CR Virus-induced atherosclerosis J Exp Med 1978 148 335 340 10.1084/jem.148.1.335 209124 PMC2184908

[B2] Wiedermann CJ Kiechl S Dunzendorfer S Schratzberger P Egger G Oberhollenzer F Willeit J Association of endotoxemia with carotid atherosclerosis and cardiovascular disease: prospective results from the Bruneck Study J Am Coll Cardiol 1999 34 1975 1981 10.1016/S0735-1097(99)00448-9 10588212

[B3] Markus HS Labrum R Bevan S Reindl M Egger G Wiedermann CJ Xu O Kiechl S Willeit J Genetic and acquired inflammatory conditions are synergistically associated with early carotid atherosclerosis Stroke 2006 37 2253 2259 10.1161/01.STR.0000236637.72124.3f 16873708

[B4] Mussa FF Chai H Wang X Yao Q Lumdsen AB Chen C *Chlamydia pneumoniae *and vascular disease: an update J Vasc Surg 2006 43 1031 1037 10.1016/j.jvs.2006.02.050 16765261

[B5] Mendall MA Goggin PM Molineaux N Levy J Toosy T Strachan D Camm AJ Northfield TC Relation of *Helicobacter pylori *infection and coronary heart disease Br Heart J 1994 71 437 439 10.1136/hrt.71.5.437 8011406 PMC483719

[B6] Blankenberg S Rupprecht H-J Blankenberg S Bickel C Kopp H Rippin G Victor A Hafner G Schlumberger W Meyer J Cytomegalovirus infection with interleukin-6 response predicts cardiovascular mortality in patients with coronary artery disease Circulation 2001 103 2915 2921 11413080 10.1161/01.cir.103.24.2915

[B7] Moutsopoulos NM Madiasanos PN Low-grade inflammation in chronic infectious diseases: paradigm of periodontal infections Ann N Y Acad Sci 2006 1088 251 264 10.1196/annals.1366.032 17192571

[B8] Beck J Garcia R Heiss G Vokonas PS Offenbacher S Periodontal disease and cardiovascular disease J Periodontal 1996 67 1123 1137 10.1902/jop.1996.67.10s.1123 29539797

[B9] Soder PO Soder B Nowak J Jogestrand T Early carotid atherosclerosis in subjects with periodontal diseases Stroke 2005 36 1195 1200 10.1161/01.STR.0000165916.90593.cb 15879347

[B10] Rupprecht HJ Blankerberg S Bickel C Rippin G Hafner G Prellwitz W Schlumberger W Meyer J AutoGene Investigators Impact of viral and bacterial infectious burden on long-term prognosis in patients with coronary artery disease Circulation 2001 104 25 31 10.1161/hc2601.091703 11435333

[B11] Stoll LL Denning GM Weintraub NL Potential role of endotoxin as a pro-inflammatory mediator of atherosclerosis Arterioscler Thromb Vasc Biol 2004 24 2227 2236 10.1161/01.ATV.0000147534.69062.dc 15472123

