# Supplementary material for: Abnormal macrophage response to microbial stimulus in a 43-year-old man with a severe form of atherosclerosis: a case report
Source: J Med Case Rep. 2010 Jun 18;4:183. doi: 10.1186/1752-1947-4-183 (PMC2909247; doi:10.1186/1752-1947-4-183)
Supplement: Additional file 1 — Materials and methods. The data provided discuss the materials and methods used in plasma preparation, clinical chemistry, cell isolation and culture, analysis of cholesterol-laden macrophages, cell protein isolation and western blotting analysis, cytokine assay, cholesterol esterification, and statistical analysis. [file 1752-1947-4-183-S1.DOC]

**Materials and Methods**

**Patient and volunteers**

Immune response to inflammatory stimuli was studied in our patient and results compared with those obtained with healthy volunteers. Due to the presence of polycythemia the patient underwent therapeutic bleeding at least once at month and the buffy coat was thus kindly provided by the Centro Trasfusionale (Azienda Ospedaliera Brotzu, Cagliari). Five healthy volunteers, aged 23-35 years, took part in the study. All subjects were non-smokers, were not taking medication, had normal ECG and echocardiogram with no evidence of atherosclerosis on carotid artery sonography. In order to exclude the possibility that potential differences in between our patient and controls may have produced a simple variability between subjects, all investigations were conducted five times, each time a new patient sample was compared with a sample from a different volunteer. Before using the blood for research purposes the patient and volunteers provided written informed consent The study was approved by the Ethical Committee of the University of Cagliari.

**Preparation of plasma**

Plasma obtained from whole blood was centrifuged at 800 RPM, red cells were removed and the supernatant centrifuged at 9000 rpm to eliminate platelets. Heat inactivation was obtained by incubating at 56°C for 30 min, afterward plasma was centrifuged, complement removed, and then filtered with 0.2 μm filter and frozen at -80°C until use.

**Clinical chemistry**

Unless otherwise stated, biochemical analysis were performed by conventional methods.

# Cell isolation and culture

Lymphomononuclear cells were separated from whole blood ^22^ and cultured at a density of 3x10^6^/ml, in RPMI 1640 supplemented with 2% heat-inactivated human AB serum, penicillin 100 U/ml, streptomycin 100 mg/ml and 2mM L-glutamine, and were allowed to adhere at 37°C in a 5% CO_2_ atmosphere for 2 hours. After removal of non adherent cells, monocytes were washed twice with pre-warmed medium and cultured with RPMI 1640 supplemented with heat-inactivated 10% AB plasma. Unless otherwise stated, in order to avoid the effect of unknown inflammatory factors present in the patient’s or volunteers plasma**,** commercially obtained AB serum (Sigma-Aldrich, Milan, Italy) was used to culture monocyte-derived macrophages. Twenty four hours later the majority of observed cells (approximately 80%) was represented by adherent monocytes with a large rounded aspect. The monolayer was washed and fresh culture medium was supplied every three days. Cells differentiated into macrophages between day 7 and day 9. In this occasion purity assessment of macrophage preparation was performed with anti CD14 FACS (FACScalibur, Beckton Dickinson), displaying a purity consistently higher than 95%.^23^ Cells were subsequently treated according to the experimental requirements as described later in this section. Supernatants were stored at -70°C and measured at the same time by the same ELISA to avoid variations during assay conditions.

*HCV-RNA and CMV-DNA detection*

RNA was extracted from each sample (serum and cell suspensions) using TRIZOL reagent^24^ (Life Technologies Inc, Gaithersburg). To evaluate the presence of HCV RNA, RNA extracts were examined by semi-nested RT-PCR.^25^ The first PCR mixture contained: 10 μM of each primer : OG 312 (5’- ACCGGGTCCTTTCTTG -3’) and OG 273 (5’- ATCATGTCCCAAGCCAT -3’) .The Second amplification mixture contained: 5 μM of each primer : OG272 (5’-TGATAGGGTGCTTGCGAG-3’ ) and OG 273 ( described previously). A 1016 bp band in agarose electrophoresis suggested a positive result for HCV RNA.

CMV -DNA from all samples was obtained by the CTAB modified method.^26^ Real time PCR was performed with a Light Cycler-RNA Amplification kit SYBR Green I (Roche Diagnostics Mannheim Germany) according to the manufacturer’s instructions. The subsequent oligos were applied: MM9 (CACGATGTTGCGCTTGTAGA ) and MM10 (GGCCCAGGGTACGGATC). This reaction amplified a fragment of 115 bp, corresponding to Glycoprotein B gene, GenBank accession n. X17403, nucleotide position 2320-2434. As positive control a synthetic 115 bp cDNA fragment manufactured by MWG-BIOTECH (www.mwg-biotech.com/html/all/index.php) and corresponding to the PCR amplicon was used.

**Cholesterol-laden macrophages**

On day 9, fresh medium was provided and 100 μg/ml of Ac-LDL (Biochemical technologies, MA, USA) added to the monolayer and incubated for up to two days to consent lipid loading.^23^

**Cell protein isolation and western blotting analysis**

The cells were lysed in a PBS buffer containing 10% SDS, 50 μg TRIS, 1 μM EDTA, pH 7.5, 50 μM DTT and a protease inhibitor cocktail, at 37°C and homogenised by a syringe fitted with an 18-gauge needle. Protein content of each sample was determined by Bradford protein assay^27^ (Bio-Rad Laboratories, Hemel Hempstead, UK). Protein samples (30μg/lane) were separated by electrophoresis (12% SDS acrylamide gel) and transferred to a PVDF membrane (Invitrogen, Milan, Italy) by standard electro-blotting procedure. The blots were pre-treated with a solution containing 5% non-fat dried milk in TBST at room temperature (50=μM TRIS-HCl, pH 7.6, 0.15 M NaCl, and 0.05% Tween-20) for at least one hour prior to addition of the primary antibodies (dilution range varying from 1:1000 to 1:3000) for human ACAT, ABCA1 and NF*k*B (Santa Cruz Biotechnology, Santa Cruz, CA). After overnight incubation the primary antibodies were removed and appropriate horseradish peroxidase-conjugated secondary antibodies (Santa Cruz Biotechnology, Santa Cruz, CA) added in a dilution range of 1:2500 to 1:5000 for at least 1h at room temperature. Proteins were detected by enhanced chemiluminescence and by exposure to X-ray film, (Amersham Pharmacia Biotech, Milano, Italy) for various times. Quantification of the protein bands was then accomplished by densitometry (Scion Imagine, NIH).

**Cytokine assay**

The levels of IL-1β, IL6, IL10, TNF-α, TGF β and INF-γ were assessed in culture supernatants from PBMC stimulated with PHA and in monocyte-derived macrophages from patient and controls. Centrifugation of the cultures was performed at 2000 rpm for 10 min to remove the cells and supernatants were frozen at -20°C until assay with a sandwich ELISA test (Immunotech SA, Marseille, France) as reported.^28^ The absorbance at 405 nm for TNF-α and at 450 nm for all others was measured with a spectrophotometer (Unidata, Rome, Italy). A standard curve was prepared by plotting the absorbance value of the standard cytokine versus the corresponding concentration (pg/ml). The range of the assay was: 5-1000 pg/ml for IL-1β, 2-1000 for IL-6, 10-1200pg/ml for TNF-α, 30-1000 pg/ml for IL-10, 50-2000 for TGF-β and TNF-α. The results were expressed in pg/ml as Mean ± Standard Error.

**Cholesterol esterification**

Cells were incubated for 4 hours in medium containing [^14^C]-oleate bound to bovine serum albumin (BSA). To prepare the oleate-BSA complex, 3.7 MBq of [^14^C]-oleic acid in ethanol (Dupont, NEN specific activity 2.035 GBq/mmol) was mixed with 1.4 mg KOH and the ethanol evaporated. PBS (1.5 ml) without Ca^2+^ and Mg^+^ containing 4.24 mg BSA (fatty acid-free, Sigma) was added and the mixture shaken vigorously. This solution was added to each well at a final concentration of 74 KBq/ml. After incubation cells were washed with ice-cold PBS and lipids extracted with acetone. Neutral lipids were separated by TLC as previously described^29^ and incorporation of [^14^C]oleate into cholesterol esters was measured. An aliquot of cell hydrolysate was processed for protein content.

**Statistical analysis**

Significant differences were determined by one- or two-way analysis of variance (ANOVA) for repeated measures (time) followed by Student's-t test where and when appropriate. Differences were considered significant if P<0.05.
